# Genome-wide identification, organization, and expression profiles of the chicken fibroblast growth factor genes in public databases and Vietnamese indigenous Ri chickens against highly pathogenic avian influenza H5N1 virus infection

**DOI:** 10.5713/ab.22.0277

**Published:** 2022-11-14

**Authors:** Anh Duc Truong, Ha Thi Thanh Tran, Nhu Thi Chu, Huyen Thi Nguyen, Thi Hao Vu, Yeojin Hong, Ki-Duk Song, Hoang Vu Dang, Yeong Ho Hong

**Affiliations:** 1 Department of Biochemistry and Immunology, National Institute of Veterinary Research, 86 Truong Chinh, Dong Da, Ha Noi 100000, Vietnam; 2 Department of Animal Science and Technology, Chung-Ang University, Anseong 17546, Korea; 3 The Animal Molecular Genetics and Breeding Center and Department of Animal Biotechnology, JeonBuk National University, Jeonju 54896, Korea

**Keywords:** Chicken, Expression Profile, Fibroblast Growth Factor, Genome-wide Analysis

## Abstract

**Objective:**

Fibroblast growth factors (FGFs) play critical roles in embryo development, and immune responses to infectious diseases. In this study, to investigate the roles of FGFs, we performed genome-wide identification, expression, and functional analyses of FGF family members in chickens.

**Methods:**

Chicken *FGFs* genes were identified and analyzed by using bioinformatics approach. Expression profiles and Hierarchical cluster analysis of the *FGFs* genes in different chicken tissues were obtained from the genome-wide RNA-seq.

**Results:**

A total of 20 *FGF* genes were identified in the chicken genome, which were classified into seven distinct groups (A–F) in the phylogenetic tree. Gene structure analysis revealed that members of the same clade had the same or similar exon-intron structure. Chromosome mapping suggested that *FGF* genes were widely dispersed across the chicken genome and were located on chromosomes 1, 4–6, 9–10, 13, 15, 28, and Z. In addition, the interactions among FGF proteins and between FGFs and mitogen-activated protein kinase (MAPK) proteins are limited, indicating that the remaining functions of FGF proteins should be further investigated in chickens. Kyoto encyclopedia of genes and genomes pathway analysis showed that *FGF* gene interacts with *MAPK* genes and are involved in stimulating signaling pathway and regulating immune responses. Furthermore, this study identified 15 differentially expressed genes (DEG) in 21 different growth stages during early chicken embryo development. RNA-sequencing data identified the DEG of FGFs on 1- and 3-days post infection in two indigenous Ri chicken lines infected with the highly pathogenic avian influenza virus H5N1 (HPAIV). Finally, all the genes examined through quantitative real-time polymerase chain reaction and RNA-Seq analyses showed similar responses to HPAIV infection in indigenous Ri chicken lines (R^2^ = 0.92–0.95, p<0.01).

**Conclusion:**

This study provides significant insights into the potential functions of FGFs in chickens, including the regulation of MAPK signaling pathways and the immune response of chickens to HPAIV infections.

## INTRODUCTION

Fibroblast growth factor (*FGF*) is a representative growth factor with potential tissue repair and regeneration effects. It consists of structurally related polypeptides that are involved in several physiological processes. These growth factors are highly conserved and found in several animal species, from nematodes and zebrafish to mice and humans [1]. *FGFs* play a role in cellular proliferation, migration, and differentiation, mitogenesis, angiogenesis, embryogenesis, and wound healing through the binding to and activation of fibroblast growth factor receptors (*FGFRs*), the main signaling pathway of which is the RAS/MAP kinase pathway [2–4]. Recently, it has been demonstrated that *FGFs* play an important role in the regeneration of damaged tissues, including skin, bone, blood vessels, muscle, cartilage, adipose tissue, tooth, tendon/ligament, and nerve, by acting on several signaling pathways, such as Wnt/beta-catenin, JAK-signal transducer and activator of transcription (STAT), and PI3K/AKT pathways [5–7]. The genes encoding FGFs have been identified in multicellular organisms ranging from invertebrates to vertebrates [8,9]. This includes the only two *FGF* genes found in *Caenorhabditis elegans*, whereas 22 *FGF* genes have been identified in humans and mice [10] and 23 FGF genes in zebrafish [11], indicating that the FGF gene family greatly expanded during the evolution of invertebrates to vertebrates. The *FGF* gene family comprises two major superfamilies based on phylogenetic tree and structural analyses. The first superfamily has one or a few archeo-FGFs to eight proto-FGFs, prototypic of the eight subfamilies. The second superfamily, which formed during the euchordate evolution, is associated with genome duplications [11]. *FGFs* play an important role in tissue repair, response to injury, and pathogenesis and as homeostatic factors in adult organisms and are also expressed and important for signal transduction in the neuronal central in adult tissue [11].

Several FGFs have been isolated and well-characterized in chickens, including *FGF1* [2], *FGF2*, *FGF4* [12,13], *FGF8* [14], *FGF12–13* [15], and *FGF19* [16]. These genes play important roles in various developmental processes of chicken embryos [16,17] and in embryonic stem cell differentiation and primordial germ cells through the activation of MAPK/extracellular signal-regulated kinases (ERK) signaling pathways [3,14]. However, the *FGF* gene family in chickens still lacks identification and information on genome structure, physicochemical properties, and phylogenetic relationships. Additionally, *FGF* gene expression under highly pathogenic avian influenza virus (HPAIV) infection is unclear.

This study identified the chromosomal locations, motif structure, genome structure, and physicochemical properties of *FGF* genes in the chicken genome. Evolutionarily conserved and class-specific residues were inferred using evolutionary trace analysis, and sequence identity dendrograms were constructed using chicken FGF nucleotide or amino acid sequences. Moreover, our results described *FGF* gene expression in different embryonic growth stages in two indigenous, genetically disparate Ri chicken lines infected with HPAIV. Our results will contribute to information on the evolution of FGFs in chickens, have possible animal breeding applications, and further clarify the mechanisms of chicken response to infectious diseases. The purposes of our study were i) to characterize all potential FGFs across the genome by performing a genome-wide comprehensive survey, ii) to estimate the potential regulatory relationship between FGFs and MAPK family proteins, and iii) to analyze transcriptional expression variation of FGFs at different growth stages of chicken embryos and iv) in response to HPAIV infection in two Vietnamese indigenous Ri chicken lines.

## MATERIALS AND METHODS

### Identification of FGF family members in chickens

The NCBI eukaryotic genome annotation resource database was searched for genome-annotated chicken, human, mouse, and zebrafish *FGF* family genes. The NCBI results were 103 for the chicken genome (*Gallus gallus*), 107 for the human genome (*Homo sapiens*), 105 for the mouse genome (*Mus musculus*), and 106 for the zebrafish genome (*Danio rerio*) and comprised several gene/protein isoforms. Because all isoforms represent one common gene/protein, we selected only the first isoform and the corresponding protein symbol for further analysis. The retrieved sequences were translated using the open reading frames (ORFs) finder (http://www.ncbi.nlm.nih.gov/gorf/gorf.html), and the predicted ORFs were verified by BLASTP against the NCBI non-redundant protein sequence database [18]. This resulted in 20 putative chicken *FGF* genes, 22 putative human and mouse *FGF* genes, and 23 putative zebrafish *FGF* genes.

### Gene structure and domain and phylogenetic analysis of chicken *FGF* genes

The gene structure was analyzed using the gene structure display server (http://gsds.cbi.pku.edu.cn/). Conserved protein motifs were identified using MEME programs as previously described [19]. The parameters of MEME for this study were as follows: optimum width, 10–60; number of repetitions, any; maximum number of motifs, 15. The isoelectric point (pI) was calculated using Compute pI/Mw (https://web.expasy.org/compute_pi/) software. The domain organization of the FGF protein family was analyzed using the SMART (http://smart.embl-heidelberg.de/) protein family (Pfam) (http://pfam.xfam.org/) and NCBI Bath Web CD-Search (https://www.ncbi.nlm.nih.gov/cdd) databases. The phylogenetic tree of the *FGF* gene family was constructed based on the protein sequence alignment of all *FGF* genes in chickens, humans, mice, and zebrafish using MEGA6 software with 1,000 bootstrap resampling [20]. *FGF* genes were classified into different groups according to the topology of the phylogenetic tree.

### Highly pathogenic avian influenza virus H5N1

The A/duck/Vietnam/QB1207/2012 (H5N1) virus, an HPAIV, isolated from a moribund domestic duck in the Quang Binh province of North Central Vietnam was used in this study [21]. The viral isolate was propagated in 10-day-old embryonated chicken eggs at 37°C for 48 hours. The allantoic fluid (AF) of the eggs was harvested, and aliquots of the AF were stored at −80°C until use, according to the World Organisation for Animal Health [22] guidelines (Chapter 3.3.4) [22]. The 50% infectious egg dose (EID50) of the influenza virus was determined as previously described [23]. Briefly, 10-fold serial dilutions of the virus were prepared in PBS, and 100 μL of each dilution was inoculated into the chorioallantoic cavities of five 10-day-old embryonated chicken eggs. The eggs were incubated at 37°C for 96 h. Harvested AF was tested for haemagglutination activity using 0.5% RBC, according to the OIE guidelines (Chapter 3.3.4) [22]. EID50/mL of virus suspension was calculated using the Reed and Muench mathematical technique [23]. The study was conducted in compliance with the institutional rules for the care and use of laboratory animals and using a protocol approved by the Ministry of Agriculture and Rural Development of Vietnam (TCVN 8402:2010 and TCVN 8400-26:2014).

### Infection of indigenous Ri chicken lines with HPAIV and high-throughput RNA-sequencing

Specific pathogen-free HPAIV-resistant and -susceptible indigenous Ri chicken lines (4 weeks of age) were purchased from the Poultry Research Centre of the National Institute of Animal Science, Vietnam. Infection studies were conducted in compliance with the institutional rules for the care and use of laboratory animals and a protocol approved by the Ministry of Agriculture and Rural Development, Vietnam (TCVN 8402:2010/TCVN 8400-26:2014). Fifteen Ri resistant and susceptible chickens per group were intranasally inoculated with AF containing 10^4^ EID50 of A/duck/Vietnam/QB1207/2012 (H5N1) in 200 μL. Fifteen uninfected Ri resistant and susceptible chickens served as the control group. Following viral infection, the chickens were checked for clinical signs of diseases, and lung samples were collected on one and three days post-infection (dpi) following the WHO Manual on Animal Influenza Diagnosis and Surveillance [22]. Total RNA was extracted from the lungs of the two Ri chicken lines and used for high-throughput RNA-Seq and data analyses, as previously described [24].

### Expression profiles of chicken *FGF* genes at different growth stages using publicly available RNA-Seq data

To explore the expression profiles of chicken *FGF* genes at different growth stages, including the oocytes, zygotes, and intrauterine embryos from Eyal-giladi and Kochav stage I (EGK.I) to EGK.X, the public high-throughput RNA-seq read databases of chicken in the Gene Expression Omnibus (accession number: GSE86592) were submitted by Hwang et al [25]. A decision-tree-based classification analysis was performed based on these class labels. Log2 trimmed mean of M-value normalized values were used to calculate a gene expression matrix and determine library size in each sample using edgeR [26]. Spearman’s correlation coefficients were calculated using a distance matrix to characterize the linear relationship between class labels and gene expression. Hierarchical cluster analysis of these genes was performed using Cluster version 4.49 (http://www.bram.org/serf/Clusters.php) and Java Treeview (http://jtreeview.sourceforge.net/). Cluster map analysis of the *FGF* genes detected between the different growth stages was performed using Euclidean distance. The p-values were calculated using right-tailed Fisher’s exact test at a 0.01 significance level.

### Pathway and interaction analysis

The cellular pathways of chicken *FGF* genes were analyzed using the Kyoto encyclopedia of genes and genomes (KEGG) pathway-mapping database against organism-specific parameters (*Gallus gallus*: gga). The interactions between chicken FGF proteins and MAPK signaling pathway genes were analyzed using the Search Tool for the Retrieval of Interacting Genes/Proteins (STRING, Version 10), as previously described [27]. The STRING database facilitates the analysis of gene/protein interactions in an organism-specific manner using commonly available sources, including the NCBI PubMed literature database.

### Quantitative real-time polymerase chain reaction analysis of FGF transcripts

For cDNA synthesis, up to 3 μg of RNA from the lung samples was treated with 1.0 unit of DNase I and 1.0 μL of 10× reaction buffer (Thermo Fisher Scientific, Waltham, MA, USA) and then incubated for 30 min at 37°C. Subsequently, 1.0 μL of 50 mM EDTA was added and heated to 65°C for 10 min to inactivate DNase I, and then the RNA was reverse transcribed using the Maxima First Strand cDNA Synthesis Kit (Thermo Fisher Scientific, USA), according to the manufacturer’s recommendations. Primers were designed using Lasergene software (DNASTAR Inc., Madison, WI, USA; Table 1), and quantitative real-time polymerase chain reaction (qRT-PCR) was performed using 2× Power SYBR Green Master Mix (Roche, Indianapolis, IN, USA) with the LightCycler 96 system (Roche, USA). Chicken glyceraldehyde-3-phosphate dehydrogenase (*GAPDH*) was used as an internal control to normalize the cytokine expression. Gene expression levels were calculated using the 2^−ΔΔ^^Ct^ method after normalization to *GAPDH* expression level [28]. All qRT-PCR experiments were performed in triplicate.

### Statistical analyses

Statistical analysis was performed using the IBM SPSS software (SPSS 25.0, IBM Corp., Armonk, NY, USA). The results are expressed as mean±standard error of three independent experiments for each group (n = 3) and were compared using Duncan’s multiple comparison method.

## RESULTS

### Identification of potential chicken *FGF* genes

We identified 20 putative *FGF* genes in the chicken genome using BLAST search and genetic analysis. General information on the 20 *FGF* family members is summarized in Table 2. According to their chromosomal positions, these genes are located on different chicken chromosomes and were named *FGF1* to *FGF23* (Table 2). Among them, chromosome 1 had four *FGF* genes, and chromosome 4 contained five *FGF* genes, chromosome 5 contained three *FGF* genes, chromosome 13 contained two *FGF* genes, and chromosomes 6, 9, 10, 15, 28, and Z had one *FGF* gene each. The ORFs of *FGF* genes ranged from 447 to 813 bp in length (Table 2). The length of the FGF proteins ranged from 148 to 270 amino acids, with predicted molecular weights ranging from 17.24 kDa to 29.51 kDa.

**Figure 1:**
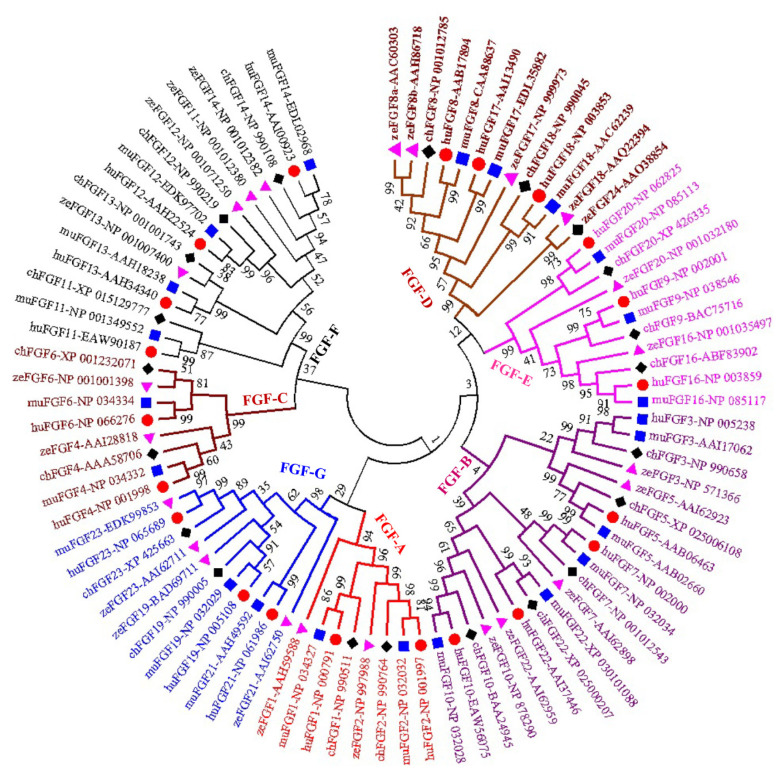
Phylogenetic analysis of chicken *FGF* genes. Full-length amino acid sequences of 20 chicken *HSP* genes were aligned via BioEdit V.7, and the phylogenetic tree was constructed in MEGA7 using the neighbor-joining method with 1,000 bootstrap replicates. *FGF*, fibroblast growth factors.

A phylogenetic tree was constructed to analyze the evolu tionary relationships among FGF proteins from chickens, humans, mice, and zebrafish. The results showed that FGF proteins could be classified into seven groups (Figure 1). Among them, two FGFs (chFGF1 and chFGF2) together with two FGF proteins from humans, mice, and zebrafish (FGF1 and FGF2) were attributed to Group A or the FGF1-subfamily (Figure 1). Group B or the FGF7-subfamily consisted of five chicken FGFs (chFGF3, chFGF5, chFGF7, chFGF10, and chFGF22) and five human, mouse, and zebrafish FGFs (FGF3, FGF5, FGF7, FGF10, and FGF22). Group C or the FGF4-subfamily consisted of two chicken FGFs (chFGF4 and chFGF6) and two human, mouse, and zebrafish FGFs (FGF4 and FGF6). Chicken FGF8 and FGF18 genes were clustered in group D or FGF8-subfamily with three genes in humans and mice (FGF8, FGF17, and FGF18) and four FGF proteins in zebrafish (FGF8, FGF17, FGF18, and FGF24). Group E or FGF9-subfamily included three proteins from chickens, humans, mice, and zebrafish (FGF9, FGF16, and FGF20). Finally, group F or FGF11-subfamily of FGF proteins included four chicken, human, mouse, and zebrafish proteins (FGF11–14) (Figure 1). The results indicate that chicken FGF proteins have a close relationship with FGF family genes in mammals and fish, suggesting similarities in the biological functions of FGF family genes.

### Gene structure, domain organization, and physicochemical analysis of chicken FGFs

**Figure 2:**
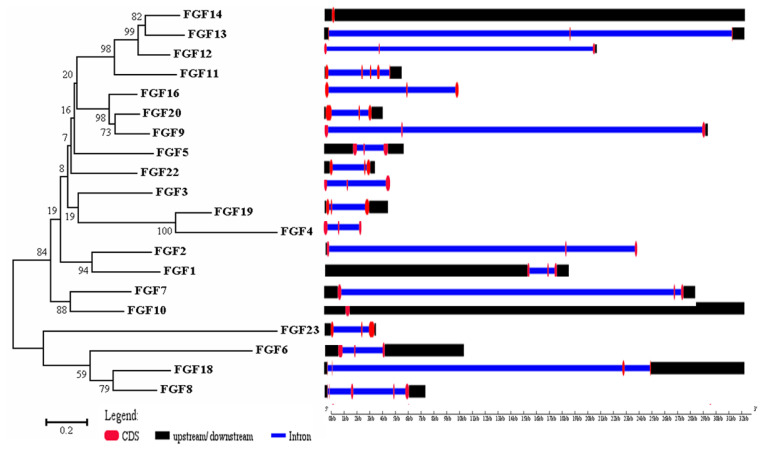
Phylogenetic analysis and gene structure of the chicken FGF genes. The unrooted neighbor-joining (NJ) tree was generated in MEGA7 with parameter settings as stated in Figure 1. The solid red boxes represent exons, black represent up/downstream genes, and green boxes represent introns. *FGF*, fibroblast growth factors.

**Figure 3:**
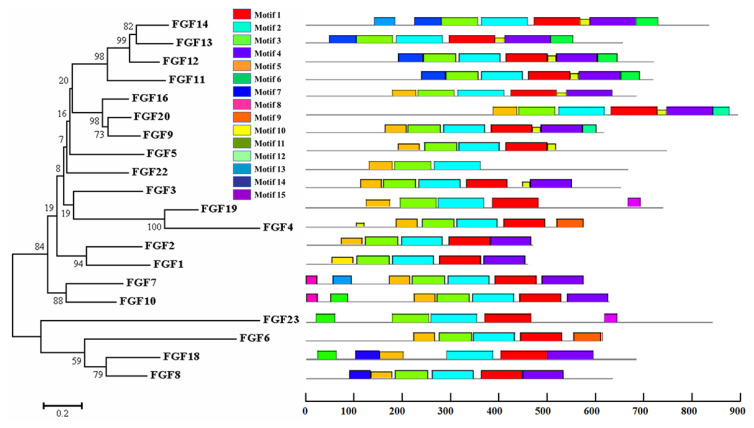
Phylogenetic analysis and conserved motifs of the chicken *FGF* genes identified using MEME (http://meme-suite.org/). The unrooted neighbor-joining (NJ) tree was generated in MEGA7 with parameter settings, as stated in Figure 1. The grey lines represent the non-conserved sequences, and each motif is indicated by a colored box numbered at the bottom. The lengths of the motifs in each protein are proportional. *FGF*, fibroblast growth factors.

The physicochemical parameters indicated that the pI ranged from 6.6 to 11.86, with most FGFs being basic except for chFGF1, chFGF9, and chFGF19, which are acidic (Table 2). We used the genome annotation file to analyze the gene structure of *FGF* genes in chickens to improve the understanding of the evolutionary conservation of the genes of this family. The exon-intron structure of each chicken *FGF* gene is shown in Figure 2. Based on the number of introns, genes could be divided into four models: model 1 with one exon and no introns, included two genes (*FGF10* and *FGF14*); model 2, containing three exons and two introns, had 15 FGF genes (*FGF1*, *FGF12*, *FGF13*, *FGF16*, *FGF19*, *FGF2*, *FGF20*, *FGF22*, *FGF23*, *FGF3*, *FGF4*, *FGF5*, *FGF6*, *FGF7*, and *FGF9*). *FGF8* and *FGF18*, were assigned to model 3 with four exons and three introns. Finally, chicken *FGF11* was assigned to model 4, which contained five exons and four introns (Figure 1 and Table 2). Conserved motif analysis was performed to understand the functional diversification of the chicken *FGF* gene family (Figure 3 and Table 3) by searching for 15 putative motifs in each gene (Figure 3 and Table 3). In general, the *FGF* gene family from the same group shared similar motifs. The sequences of the conserved motifs ranged from 6 to 29 amino acids, and all *FGF* proteins contained at least three motifs. Motif 2 was present in all chicken *FGF* genes, and motifs 1 and 3 were present in 19 chicken *FGF* genes (Figure 3).

### Potential regulatory relationship between chicken FGFs and MAPK protein

**Figure 4:**
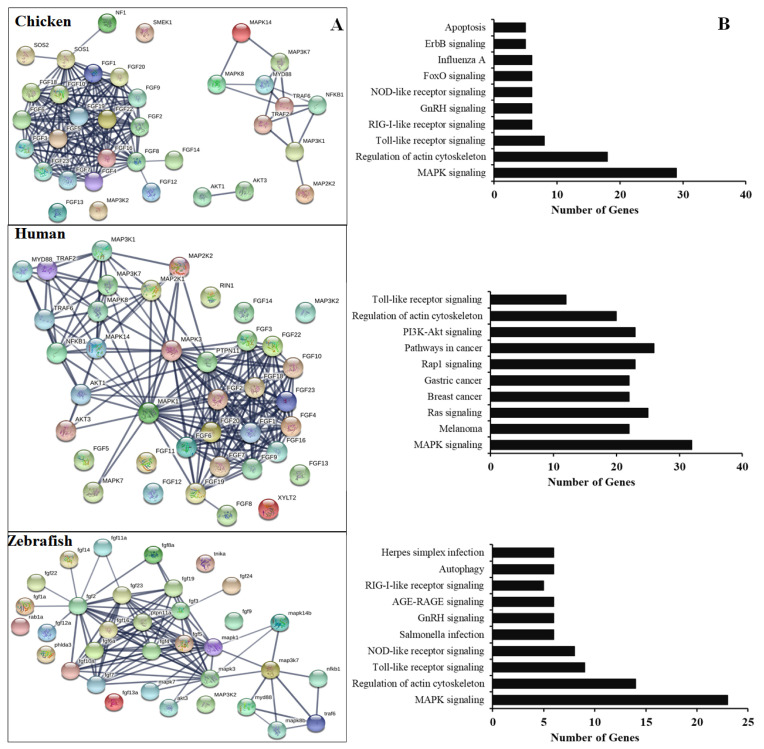
(A) Protein-protein interactions of chicken FGFs and MAPK signaling proteins. Evidence-based medium-confidence interactions (score, 0.700) of FGFs with other FGFs and MAPK signaling proteins were identified using the STRING program (https://string-db.org/). (B) Cellular pathways of proteins encoded by FGF and MAPK molecular genes. The KEGG pathway mapping database determined the cellular pathways of 20 proteins of FGFs and MAPK signaling (https://www.genome.jp/kegg/). FGF, fibroblast growth factors; MAPK, mitogen-activated protein kinase; KEGG, Kyoto encyclopedia of genes and genomes.

To further understand the functional *FGF* genes in chickens, we performed an interaction analysis between FGF and MAPK family proteins and compared chickens, humans, and zebrafish. The results showed a limited interaction between FGF proteins and MAPK family proteins in chicken, with only SOS1 protein showing any interaction with FGF family proteins. The predicted significant interactions among FGFs and between FGFs and MAPK subunits were merely derived from text-mining data and experimental evidence and lacked gene fusions (Figure 4, bottom). Therefore, co-expression and co-functional-based studies among FGFs and between FGFs and MAPK subunit signaling proteins should be conducted to provide experimental evidence. In contrast, similar interactions for human genes were derived from text-mining data, experimental evidence, gene fusions, co-expression, and co-function (Figure 4A, middle). More research was conducted on gene fusion, co-expression, and co-function between FGFs and MAPK family proteins in zebrafish than in chickens (Figure 4A, bottom). The results indicate that research on FGF function and co-expression and co-function between FGFs and MAPK family proteins in chickens should be performed in the future.

### Expression profiles of *FGF* genes at different growth stages in chickens

**Figure 5:**
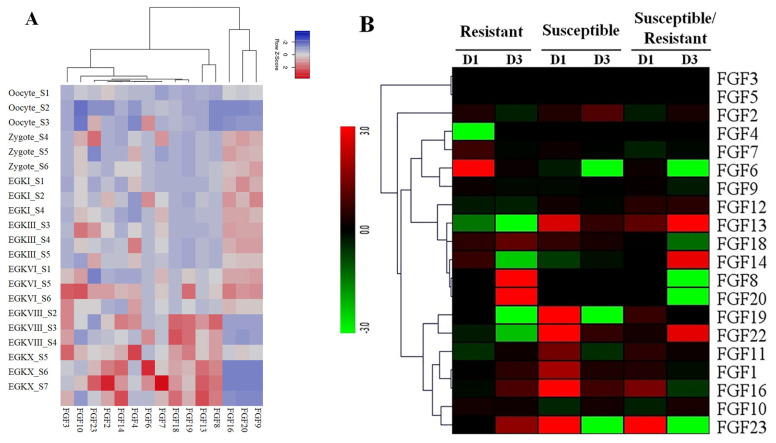
(A) Expression profiles of FGF genes in the 21 different chicken early embryo growth stages, including the oocytes, zygotes, and intrauterine embryos from Eyal-giladi and Kochav stage I (EGK.I) to EGK.X. Different colors correspond to log2 transformed values. Red or blue indicates higher or lower relative abundance of each transcript in each sample, respectively. (B) Expression profiles of FGF genes in two indigenous Ri chicken lines infected with 104 EID50 of HPAIV compared to respective controls. The heatmap was generated from a hierarchical analysis of the 20 FGF genes that showed significant changes in expression in the HPAIV-afflicted chicken lines. The genes showed significant differences in expression (p<0.01, fold change ≥2). The genes shown in red were upregulated, and those in green were downregulated in the two chicken lines infected with HPAIV. Hierarchical clusters of genes and samples were drawn based on Pearson’s correlation analyses. FGF, fibroblast growth factors; HPAIV, highly pathogenic avian influenza virus H5N1.

To obtain more insight into the temporal and spatial expression patterns of chicken *FGF* genes during embryonic development, chicken RNA-seq data from GSE86592 [25] were used to explore the expression profiles of *FGF* genes in the 21 different growth stages of chicken early embryos, as shown in Figure 5A. The 5/20 *FGF* genes were not expressed in early chicken embryos at 21 different growth stages. The results showed that 15 out of 20 *FGF* genes were expressed in all 21 growth stages of early chicken embryos and were differentially expressed in early chicken embryos. For example, *FGF9*, *FGF16*, and *FGF20* were upregulated in Zygote S4–6, EGKI, EGKIII_S3–5, EGKVI_S1, S5, and S6 stages but were downregulated in all remaining early chicken embryo growth stages (Figure 5A). Moreover, *FGF* genes in some groups were upregulated and downregulated at different stages, such as *FGF8*, *FGF13*, *FGF18*, and *FGF19*, which were upregulated at EGKVIII_S2–4 and EGKX_S5–7 but were downregulated at another chicken early embryo growth stage (Figure 5A). These results indicated that *FGF* genes were diffirentially expressed in chicken early embryo growth stages and suggested the role of *FGF* genes in early chicken embryo growth stages in responses to environmental conditions (Figure 5A).

### Expression profiles of *FGF* genes in Vietnamese indigenous Ri chickens infected with HPAIV

The results of transcriptome sequencing analysis against HPAIV infection and control in the lungs of the two indigenous Ri chicken lines are shown in Figure 5B. Our data showed that 20/20 *FGF* genes were expressed in the lungs of the two indigenous Ri chicken lines (Figure 5B). Data analysis revealed that most chicken *FGF* genes exhibited broad expression patterns (Figure 5B). At 1 dpi, six genes in the indigenous Ri resistant line (*FGF2*, *FGF6*, *FGF7*, *FGF14*, *FGF18*, and *FGF19*) and nine genes in the susceptible line (*FGF2*, *FGF13*, *FGF18*, *FGF19*, *FGF22*, *FGF1*, *FGF11*, *FGF16*, and *FGF23*) were upregulated in the lungs compared to the respective controls (Figure 5B). At 3 dpi, seven genes were significantly upregulated in the resistant line (*FGF18*, *FGF8*, *FGF20*, *FGF1*, *FGF11*, *FGF16*, and *FGF23*), and five genes (*FGF2*, *FGF13*, *FGF22*, *FGF1*, and *FGF16*) were significantly upregulated in the susceptible line (Figure 5B). When comparing susceptible and resistant chicken lines infected with HPAIV, we found three significantly upregulated genes (*FGF13*, *FGF16*, and *FGF23* with log 2-fold change ranked 1.13 to 5.18) at 1 dpi. In comparison, three genes (*FGF22*, *FGF13*, and *FGF14* with log fold change ranked 2.07 to 8.63) were significantly upregulated, and five genes (*FGF18*, *FGF20*, *FGF6*, *FGF23*, and *FGF8* with log fold change ranked 1.27 to 8.10) were significantly downregulated at 3 dpi. The other *FGF* genes were significantly expressed after HPAIV infection in the two indigenous Ri chicken lines (Figure 5B). The results demonstrated that the expression of *FGF* genes was significantly related to HPAIV infection in the two indigenous Ri chicken lines.

### Validation of RNA-Seq results by quantitative real-time polymerase chain reaction

**Figure 6:**
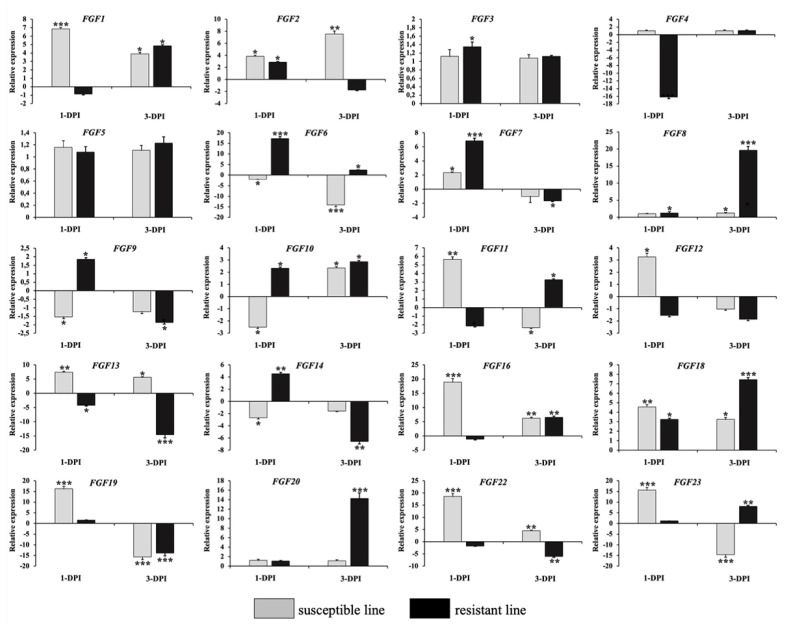
Validation of 20 significantly differentially expressed chicken *FGF* genes using qRT-PCR. The two indigenous Ri chicken lines were infected with 104 EID50 of HPAIV, and lung samples were isolated from chickens at 1 and 3 dpi. Data are expressed as mRNA levels normalized against the GAPDH mRNA level with triplicate determination performed with pooled samples from five chickens. Significant differences in mRNA expression levels between two chicken lines are indicated as follows: * p<0.05, ** p<0.01, and *** p<0.001. Error bars indicate the SEM of technical replicates that were analyzed in triplicate. *FGF*, fibroblast growth factors; qRT-PCR, quantitative real-time polymerase chain reaction; HPAIV, highly pathogenic avian influenza virus H5N1; GAPDH, glyceraldehyde-3-phosphate dehydrogenase.

**Figure 7:**
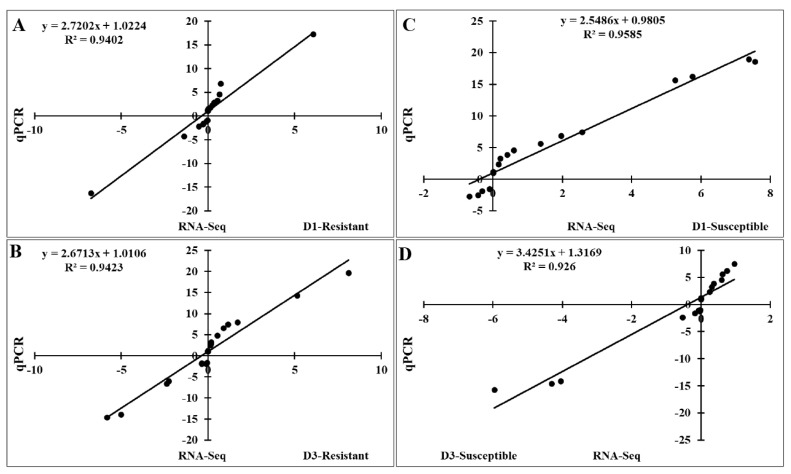
Significant correlations between expression of qRT-PCR and RNA-Seq using the lung samples of two indigenous Ri chicken lines infected with HPAIV. The correlation between RNA-Seq and qRT-PCR data in the resistant line at 1 dpi (A) and 3 dpi (B) and in the susceptible line at 1 dpi (C) and 3 dpi (D). qRT-PCR, quantitative real-time polymerase chain reaction; HPAIV, highly pathogenic avian influenza virus H5N1.

To validate the RNA-Seq results, we performed qRT-PCR to analyze the expression of 20 *FGF* genes in the lungs of two chicken lines infected with HPAIV compared to the respective uninfected controls (Figure 6). Compared to the control groups, the resistant and susceptible lines showed that the mRNA expression of 7/20 and 10/20 FGFs was significantly upregulated at 1 dpi, respectively. At 3 dpi, 9/20 and 5/20 *FGF* mRNAs were considerably upregulated in the resistant and susceptible lines, respectively. In contrast, the expression of 3/20 *FGF* mRNAs was dramatically downregulated at 1 dpi in both lines, and 5/20 and 3/20 *FGF* mRNA were significantly downregulated at 3 dpi in the resistant and susceptible lines, respectively (Figure 6). The other *FGF* genes showed little changes in expression levels after HPAIV infection in the two indigenous Ri chicken lines. The expression trends of the qRT-PCR findings were consistent with the RNA-Seq results (correlation R^2^ = 0.9402 and 0.9423 for HPAIV-infected resistant lines at 1 and 3 dpi, respectively and correlation R^2^ = 0.9585 and 0.9260 for HPAIV-infected susceptible lines at 1 and 3 dpi, respectively), as shown in Figure 7.

## DISCUSSION

FGFs play an important role in various developmental processes of chicken embryos [16,17] and in embryonic stem cell differentiation and primordial germ cells through the activation of MAPK/ERK signaling pathways [3,14]. Several chicken FGFs have been identified and functionally characterized, including FGF1 [2], FGF2, FGF4 [12,13], FGF8 [14], FGF12–13 [15], and FGF19 [16].

This study observed that the distribution of *FGF* genes in chicken was dispersed over 10/36 chromosomes, similar to the results reported in humans [29] and common carp [30], suggesting that the *FGF* genes of chicken are conserved with those of other species. Computational analyses assessing the physicochemical properties of proteins encoded by gene families play an important role in understanding the functions of the proteins *in vitro*. In this study, the pI of chicken FGF proteins revealed that 3 were acidic and 17 were basic. These observations might indicate functional differences in chicken FGF proteins compared to other members, as similar findings might suggest different roles.

A bioinformatics approach was used to identify conserved residues crucial to the function or structure of proteins and related proteins between homologous sequences in the species. This study identified the closest genes in the same family or subfamily with similar exon/intron structures and intron numbers (Figure 1). The phylogenetic tree for the *FGF* family showed that the number of exons/introns was similar in certain sister pairs. Motif analysis revealed that the type, order, and number of motifs were similar in protein sequences within the same family/subfamily but differed from those of proteins in other families and subfamilies (Figure 3).

In chicken, the *FGF* gene/protein family plays an important role in immune response and cell death, especially during the development of chicken embryos. It also plays an important role in embryonic stem cell differentiation and primordial germ cells [3,14,16,17]. Several studies have demonstrated that the mammalian FGF proteins are associated with and induce the expression of several genes, such as apoptosis and immune-related genes, growth factors, cytokines, by activation of MAPK family proteins, STATs, or nuclear factor kappa B (NF-κB) subunits [3,4,7]. Our analysis demonstrated high interactions among chicken *FGF* family genes/proteins but limited interaction between *FGF* genes/proteins with MAPK/NF-κB signaling pathway proteins. In contrast, the high interaction between human FGF family genes/proteins and MAPK/NF-κB signaling pathway components and the function of *FGF* genes in humans to control the immune response to pathogens have been investigated [2–4,7]. The results indicated that the function of FGF protein inactivation and the association of FGFs with molecular signaling pathway proteins and immune system regulation in chickens should be investigated and evaluated *in vitro* and *in vivo*. Moreover, the remaining FGF proteins have not been identified and functionally characterized in chickens and should be investigated in a future study. Furthermore, KEGG databases showed that the chicken *FGF* gene family is involved in the immune response to the pathogen or signaling pathways similar to those in humans and zebrafish. The results indicated that the main functions of FGFs in chickens are the regulation of apoptosis, development of chicken embryos, and immune response to the pathogen (Figure 4).

Chicken RNA-Seq data from public databases were ex plored to dissect the expression profiles of *FGF* genes. Our analysis indicated that five *FGF* genes (*FGF1*, *FGF5*, *FGF11*, *FGF12*, and *FGF22*) were not expressed in early chicken embryos in 21 different growth stages, while 15 were expressed in all 21 growth stages, indicating that *FGF* genes were differentially expressed in early chicken embryos. Previous reports indicated that *FGF* family genes such as *FGF1* [2], *FGF2* and *FGF4* [12,13], *FGF8* [14], *FGF12–13* [15], and *FGF19* [16] play important roles in the development of embryonic chickens [13,14,31–33]. Our results showed that the different *FGF* gene families play important roles in the different growth stages of early chicken embryos. Moreover, our results also demonstrated that 20 *FGF* genes were expressed in the HPAIV-infected lungs of two indigenous Ri chicken lines (resistant and susceptible). Recent research has indicated that *FGF* genes such as *FGF23*, *FGF2*, *FGF1–4*, and *FGF7* play important roles in the immune response to pathogenic and environmental conditions [34–38]. Our results are the first to analyze the response of the *FGF* genes to HPAIV infection in indigenous chicken lines. These results suggest that the *FGF* gene family plays an essential role in the development of chicken embryos and immune responses in adult chickens. However, the function of the *FGF* gene family has not been investigated in chickens. Aspects such as the expression and regulation of *FGF* genes in pathogen infection and the regulation of *FGF* genes in the development of chicken embryos, cytokine expression, apoptosis, or immune system through regulation of signaling pathways, immune pathway, apoptosis pathway, or development pathway in chickens, such as in the response to infectious diseases, NF-κB signaling pathway, JAK-STAT signaling pathway, toll like receptor pathway, apoptosis pathway, cell cycle, or B-T cell signaling pathway should be addressed. Furthermore, the remaining FGF proteins have not been identified and characterized in chickens. This should be done in a future study, especially investigating the function of *FGF* family genes in response to infectious diseases.

In conclusion, 20 *FGF* gene families were identified in this genome-wide survey of the chicken genome. According to the phylogenetic tree analysis, *FGF* genes were classified into seven groups. Protein–protein interactions with proteins from MAPK and NF-κB signaling pathway were low and were not identified and characterized in chickens, which should be investigated in future studies. In contrast, KEGG analysis indicated that the *FGF* gene family mainly regulates the immune response to pathogens through several signaling pathways. Finally, the *FGF* gene family was differentially expressed in chicken early embryos and in two HPAIV-infected Vietnamese indigenous Ri chicken lines.

## Figures and Tables

**Table 1:** Primers used for quantitative real-timepolymerase chain reaction validation

Primers	F/R	Nucleotide sequence (5′-3′)	Accession number
GAPDH	Forward	TGCTGCCCAGAACATCATCC	NM_204305
	Reverse	ACGGCAGGTCAGGTCAACAA	
FGF1	Forward	TCGTCGGGCTGAAGAAAAAC	NM_205180
	Reverse	ACCGGCAATGGGAGGAAG	
FGF2	Forward	TCGAGCGCTTGGAATCTAATAA	NM_205433
	Reverse	GTTTTTGGTCCGGGCTTGTA	
FGF3	Forward	AACGGCACCTTGGAGAAAAA	NM_205327
	Reverse	AAGCCCTTGATAGCGACGAT	
FGF4	Forward	CCACAGCGAGAACCGATACA	NM_001031546
	Reverse	TCATGGCCACGAAGAGTCC	
FGF5	Forward	CCACCGCCACCCAAGAA	XM_025150340
	Reverse	GCCACTACCCAAAGCGAAACT	
FGF6	Forward	GAAGCCAACACTGAAACCACA	XM_001232070
	Reverse	CTGCCAAACTTACCACACCTG	
FGF7	Forward	GCTTCTGCAAAATGGACA	NM_001012525
	Reverse	AGAGGAAGAAAGTGGGATGC	
FGF8	Forward	CTCGTGCGCACCTACCAG	NM_001012767
	Reverse	CTCGGCCATCGCATTGA	
FGF9	Forward	CACCAGGCAAGACCACAGC	NM_204399
	Reverse	CTCCCCTTTCTCATTCATTCC	
FGF10	Forward	GGCGATCTGTCCCCTGAGT	NM_204696
	Reverse	CCGGTGGTTGCTGCTTCT	
FGF11	Forward	CCCCACCGCCTTTTCTATG	XM_015274291
	Reverse	CGTGGCCTTATTGCTATTGGT	
FGF12	Forward	CAAGGACGAAAACAGCGACTAC	NM_204888
	Reverse	TGCCTTCACCCCTTGGATT	
FGF13	Forward	CTGGCCGGGGTTGGTATT	NM_001001743
	Reverse	GCTGCAGGTTTATTTTTCTTCA	
FGF14	Forward	GCACCCCGATGGAAGTCTC	NM_204777
	Reverse	TTTACACCCTGGATAGCAACAA	
FGF16	Forward	CGCTTCGGGATTTTGGAGT	NM_001044650
	Reverse	CGTTCATGCCGAGGTAGAGTC	
FGF18	Forward	TGTTGCCGAGGAGAATGTAGAT	NM_204714
	Reverse	TCCCGCTTGTCCTGCTGTA	
FGF19	Forward	GGCTGCGGCACCTCTACA	NM_204674
	Reverse	TGCGGGCTCTGGCTACC	
FGF20	Forward	GATTCTGGGCGGCGATACT	XM_426335
	Reverse	AGGTCTGGGCAGGAAATGTGT	
FGF22	Forward	CCACCCGCTTCTTCCTGA	XM_025144439
	Reverse	TGGCCACGACTCCGACAC	
FGF23	Forward	TCAGATACCGAACAGCCACTAA	XM_425663
	Reverse	TGCAGCCTTCAGGATACAGA	

GAPDH, glyceraldehyde-3-phosphate dehydrogenase; FGF, fibroblast growth factors.

**Table 2:** Summary information of the chicken *FGF* genes

No.	Gene	Ensembl gene ID	Accession number		Gene		Protein			Genome			
			
			mRNA	Protein	mRNA (bp)	CDS (bp)	Amino acids	Mol (kDa)	pI	Location	Gene length (bp)	Exon	Intron
1	*FGF1*	ENSGALG00000031255	NM_205180	NP_990511.1	1,419	468	155	17.191	6.96	chr13:17962472-17978333	19,000	3	2
2	*FGF2*	ENSGALG00000011835	NM_205433	NP_990764	475	447	148	17.243	9.59	chr4:53381733-53603062	24,297	3	2
3	*FGF3*	ENSGALG00000026853	NM_205327	NP_990658	663	663	220	22.910	10.87	chr5:17872480-17877619	5,139	3	2
4	*FGF4*	ENSGALG00000007562	NM_001031546	AAA58706	585	585	194	21.642	10.37	chr5:17843043-17845980	2,937	3	2
5	*FGF5*	ENSGALG00000045521	XM_025150340	XP_025006108	2,030	756	251	27.196	11.86	chr4:45319310-45324873	6,199	3	2
6	*FGF6*	ENSGALG00000017287	XM_001232070	XP_001232071	7,806	621	206	22.862	9.28	chr1:73398599-73411082	10,817	3	2
7	*FGF7*	ENSGALG00000028158	NM_001012525	NP_001012543	681	585	194	22.513	9.53	chr10:10988961-11016023	28,931	3	2
8	*FGF8*	ENSGALG00000007706	NM_001012767	NP_001012785	645	645	214	22.273	10.26	chr6:23669485-23675847	7,856	4	3
9	*FGF9*	ENSGALG00000025748	NM_204399	BAC75716	819	627	208	23.382	6.6	chr1:179686486-179717720	29,876	3	2
10	*FGF10*	ENSGALG00000014872	NM_204696	BAA24945	950	639	212	23.631	9.7	chrZ:13972676-14032776	62,068	1	0
11	*FGF11*	ENSGALG00000047749	XM_015274291	XP_015129777	1,167	732	243	26.264	8.56	chr15:113068-116313	6,007	5	4
12	*FGF12*	ENSGALG00000036971	NM_204888	NP_990219	825	732	243	27.324	9.92	chr9:13490726-13696621	189,270	3	2
13	*FGF13*	ENSGALG00000006508	NM_001001743	NP_001001743	841	600	199	22.185	9.29	chr4:4750151-4964186	215,316	3	2
14	*FGF14*	ENSGALG00000016866	NM_204777	NP_990108	1,129	762	253	28.509	9.7	chr1:144408606-144791819	390,207	1	0
15	*FGF16*	ENSGALG00000007806	NM_001044650	ABF83902	624	624	207	23.650	9.22	chr4:12790679-12801083	10,404	3	2
16	*FGF18*	ENSGALG00000002203	NM_204714	NP_990045	624	624	207	20.971	9.82	chr13:3919089-4012490	65,681	4	3
17	*FGF19*	ENSGALG00000028376	NM_204674	NP_990005	2,262	675	224	22.289	6.96	chr5:17813608-17818546	4,938	3	2
18	*FGF20*	ENSGALG00000013663	XM_426335	XP_426335	1,805	813	270	29.510	10	chr4:63267128-63271376	4,564	3	2
19	*FGF22*	ENSGALG00000041822	XM_025144439	XP_025000207	1,402	615	204	20.860	11.78	chr28:2814161-2817295	3,955	3	2
20	*FGF23*	ENSGALG00000027791	XM_425663	XP_425663	1,354	765	254	28.752	7.69	chr1:73425404-73429018	3,998	3	2

FGF, fibroblast growth factors; CDS, coding sequence; pI, isoelectric point.

**Table 3:** Fifteen different motifs commonly observed in chicken FGF proteins

Motif	Motif sequence	Sites	Width	E-value
MEME-1	FTEECKFKERVEENNYNTYASAKYRHQYS	19	29	5,00E−190
MEME-2	JISVAVGVVAIKGVKSGLYLAMNKKGKLY	20	29	5,00E−189
MEME-3	YHLQILPDGRVDGTREENSPSTJ	19	23	4,10E−104
MEME-4	ALNKKGRPRKGNRTKKHQKAAHFLPRPVD	14	29	1,10E−94
MEME-5	HLGGILRRRRLYCRT	15	15	1,10E−30
MEME-6	VAMYREPSLHDIGE	4	14	2,00E−10
MEME-7	PEPQLKGIVTKLYSRQG	4	17	1,90E−06
MEME-8	MCKWILTW	2	8	1,30E−02
MEME-9	KKGNKVSPAMTVTHFLPRI	2	19	7,00E−02
MEME-10	GRAWYV	10	6	8,50E−02
MEME-11	CSCLCLLFLVLC	3	12	1,50E+01
MEME-12	PEKVPELYKD	2	10	1,90E+02
MEME-13	WNIFLKGSIMLQC	2	13	2,20E+02
MEME-14	DFRQHVEEQSRVRDD	2	15	1,00E+03
MEME-15	DPLDPFGI	3	8	1,30E+03

FGF, fibroblast growth factors.

## References

[1] Olapoju SO, Adejobi OI, Le Thi X (2020). Fibroblast growth factor 21; review on its participation in vascular calcification pathology. Vascul Pharmacol.

[2] Forough R, Weylie B, Patel C (2005). Role of AKT/PKB signaling in fibroblast growth factor-1 (FGF-1)-induced angiogenesis in the chicken chorioallantoic membrane (CAM). J Cell Biochem.

[3] Choi JW, Kim S, Kim TM (2010). Basic fibroblast growth factor activates MEK/ERK cell signaling pathway and stimulates the proliferation of chicken primordial germ cells. PLoS One.

[4] Wu YF, Zhang DD, Liu SY (2018). C-Type natriuretic peptide dampens fibroblast growth factor-23 expression through MAPK signaling pathway in human mesangial cells. J Interferon Cytokine Res.

[5] Wang X, Zhu Y, Sun C (2017). Feedback activation of basic fibroblast growth factor signaling via the wnt/beta-catenin pathway in skin fibroblasts. Front Pharmacol.

[6] Li S, Guo X, Zhang T (2017). Fibroblast growth factor 21 ameliorates high glucose-induced fibrogenesis in mesangial cells through inhibiting STAT5 signaling pathway. Biomed Pharmacother.

[7] Yang PH, Zhu JX, Huang YD (2016). Human basic fibroblast growth factor inhibits tau phosphorylation via the PI3K/Akt-GSK3beta signaling pathway in a 6-hydroxydopamine-induced model of Parkinson’s disease. Neurodegener Dis.

[8] Borland CZ, Schutzman JL, Stern MJ (2001). Fibroblast growth factor signaling in Caenorhabditis elegans. Bioessays.

[9] Duszynski RJ, Topczewski J, LeClair EE (2013). Divergent requirements for fibroblast growth factor signaling in zebrafish maxillary barbel and caudal fin regeneration. Dev Growth Differ.

[10] Zhan DC, Shen YS, Zhao YR, Meng FJ (2019). Efficacy and safety of basic fibroblast growth factor in the treatment of burns: Protocol for a systematic review and meta-analysis of randomized controlled trials. Medicine (Baltimore).

[11] Gallegos TF, Kamei CN, Rohly M, Drummond IA (2019). Fibroblast growth factor signaling mediates progenitor cell aggregation and nephron regeneration in the adult zebrafish kidney. Dev Biol.

[12] Schofer C, Frei K, Weipoltshammer K, Wachtler F (2001). The apical ectodermal ridge, fibroblast growth factors (FGF-2 and FGF-4) and insulin-like growth factor I (IGF-I) control the migration of epidermal melanoblasts in chicken wing buds. Anat Embryol (Berl).

[13] Dono R, Zeller R (1994). Cell-type-specific nuclear translocation of fibroblast growth factor-2 isoforms during chicken kidney and limb morphogenesis. Dev Biol.

[14] Wang M, Zhang C, Huang C (2018). Regulation of fibroblast growth factor 8 (FGF8) in chicken embryonic stem cells differentiation into spermatogonial stem cells. J Cell Biochem.

[15] Munoz-Sanjuan I, Simandl BK, Fallon JF, Nathans J (1999). Expression of chicken fibroblast growth factor homologous factor (FHF)-1 and of differentially spliced isoforms of FHF-2 during development and involvement of FHF-2 in chicken limb development. Development.

[16] Kurose H, Bito T, Adachi T, Shimizu M, Noji S, Ohuchi H (2004). Expression of Fibroblast growth factor 19 (Fgf19) during chicken embryogenesis and eye development, compared with Fgf15 expression in the mouse. Gene Expr Patterns.

[17] Frohns F, Mager M, Layer PG (2009). Basic fibroblast growth factor increases the precursor pool of photoreceptors, but inhibits their differentiation and apoptosis in chicken retinal reaggregates. Eur J Neurosci.

[18] Kent WJ (2002). BLAT--the BLAST-like alignment tool. Genome Res.

[19] Bailey TL, Boden M, Buske FA (2009). MEME SUITE: tools for motif discovery and searching. Nucleic Acids Res.

[20] Tamura K, Stecher G, Peterson D, Filipski A, Kumar S (2013). MEGA6: molecular evolutionary genetics analysis version 6.0. Mol Biol Evol.

[21] Bui VN, Ogawa H, Trinh DQ (2014). Genetic characterization of an H5N1 avian influenza virus from a vaccinated duck flock in Vietnam. Virus Genes.

[22] OIE (2018). Chapter 3.3.4: Avian influenza (infection with avian influenza viruses) (NB: Version adopted in May 2015.

[23] Reed LJ, Muench H (1938). A simple method of estimating fifty per cent endpoints. Am J Epidemiol.

[24] Vu TH, Hong Y, Truong AD (2022). Cytokine-cytokine receptor interactions in the highly pathogenic avian influenza H5N1 virus-infected lungs of genetically disparate Ri chicken lines. Anim Biosci.

[25] Hwang YS, Seo M, Lee BR (2018). The transcriptome of early chicken embryos reveals signaling pathways governing rapid asymmetric cellularization and lineage segregation. Development.

[26] Trapnell C, Williams BA, Pertea G (2010). Transcript assembly and quantification by RNA-Seq reveals unannotated transcripts and isoform switching during cell differentiation. Nat Biotechnol.

[27] Szklarczyk D, Morris JH, Cook H (2017). The STRING database in 2017: quality-controlled protein-protein association networks, made broadly accessible. Nucleic Acids Res.

[28] Livak KJ, Schmittgen TD (2001). Analysis of relative gene expression data using real-time quantitative PCR and the 2(−Delta Delta C(T)) method. Methods (San Diego, Calif. ).

[29] Ruiz-Narvaez EA, Haddad SA, Lunetta KL (2016). Gene-based analysis of the fibroblast growth factor receptor signaling pathway in relation to breast cancer in African American women: the AMBER consortium. Breast Cancer Res Treat.

[30] Jiang L, Zhang S, Dong C (2016). Genome-wide identification, phylogeny, and expression of fibroblast growth genes in common carp. Gene.

[31] Bagchi M, Ireland M, Katar M, Maisel H (2001). Heat shock proteins of chicken lens. J Cell Biochem.

[32] Munaim SI, Klagsbrun M, Toole BP (1988). Developmental changes in fibroblast growth factor in the chicken embryo limb bud. Proc Natl Acad Sci USA.

[33] Consigli SA, Joseph-Silverstein J (1991). Immunolocalization of basic fibroblast growth factor during chicken cardiac development. J Cell Physiol.

[34] Mattoo RL (2014). The roles of fibroblast growth factor (FGF)-23, alpha-klotho and furin protease in calcium and phosphate homeostasis: a mini-review. Indian J Clin Biochem.

[35] Chen G, Qiu H, Ke S, Hu S, Yu S, Zou S (2013). The fibroblast growth factor receptor 2-mediated extracellular signal-regulated kinase 1/2 signaling pathway plays is important in regulating excision repair cross-complementary gene 1 expression in hepatocellular carcinoma. Biomed Rep.

[36] Jeong W, Lee J, Bazer FW, Song G, Kim J (2016). Fibroblast growth factor 4-induced migration of porcine trophectoderm cells is mediated via the AKT cell signaling pathway. Mol Cell Endocrinol.

[37] Charoenngam N, Rujirachun P, Holick MF, Ungprasert P (2019). Oral vitamin D3 supplementation increases serum fibroblast growth factor 23 concentration in vitamin D-deficient patients: a systematic review and meta-analysis. Osteoporos Int.

[38] Tsai WC, Wu HY, Peng YS (2018). Effects of lower versus higher phosphate diets on fibroblast growth factor-23 levels in patients with chronic kidney disease: a systematic review and meta-analysis. Nephrol Dial Transplant.

